# The Pharmacokinetics of Fucoidan after Topical Application to Rats

**DOI:** 10.3390/md17120687

**Published:** 2019-12-06

**Authors:** Olga N. Pozharitskaya, Alexander N. Shikov, Ekaterina D. Obluchinskaya, Heikki Vuorela

**Affiliations:** 1 Federal State Budgetary Scientific Institution of Murmansk Marine Biological Institute, Kola Scientific Center of the Russian Academy of Sciences (MMBI KSC RAS), Vladimirskaya, 17, 183010 Murmansk, Russia; olgapozhar@mail.ru (O.N.P.); okaterine@yandex.ru (E.D.O.); 2 St. Petersburg State Chemical Pharmaceutical University, Prof. Popov, 14, 197376 Saint-Petersburg, Russia; 3 Drug Research Program, Division of Pharmaceutical Biosciences, Faculty of Pharmacy, University of Helsinki, P.O. Box 56 (Viikinkaari 5E), FI-00014 Helsinki, Finland; heikki.vuorela@helsinki.fi

**Keywords:** fucoidan, topical application, pharmacokinetics, ointment, skin, striated muscle

## Abstract

Fucoidan, a fucose-rich polysaccharide from brown algae, has been used for transdermal formulations targeting inflammatory skin conditions, for the treatment of thrombosis, vascular permeability diseases, subcutaneous wounds, and burns. However, the pharmacokinetics of fucoidan after topical application has not been described. In this study, an ointment (OF) containing 15% fucoidan was topically applied to rats at the doses of 50–150 mg/g. The anti-Xa activity was selected as the biomarker, and the amidolytic assay method was validated and applied for pharmacokinetic studies of fucoidan. Fucoidan in OF penetrated the skin and distributed into the skin, striated muscle, and plasma with AUC_0–48_ = 0.94 μg·h/g, 2.22 μg·h/g, and 1.92 µg·h/mL, respectively. The longest half-life for fucoidan was observed in plasma, then in striated muscle and skin. It was found that the pharmacokinetics of fucoidan after topical OF application was linear, in the range of 50–150 mg/kg. No accumulation of fucoidan in plasma was observed after repeated topical applications of 100 mg/kg during five days. Our results support the rationality of topical application of formulations with fucoidan.

## 1. Introduction

Fucoidans are one of the main fucose-rich polysaccharides isolated from *Ascophyllum* spp., *Fucus* spp., *Laminaria* spp., *Undaria* spp., and other brown algae. An attractive array of biological activities of fucoidan has been reported for its immune modulation, inhibition of tumor cells, blood lipid-reduction, treatment of age-related macular degeneration, antioxidant activity, antimicrobial properties, anti-viral vaccine adjuvant, etc. [1,2,3,4,5,6]. 

Topical application of fucoidan exerts an anti-inflammatory effect on the skin. Fucoidan inhibits the expression of ultraviolet (UV) matrix metalloprotease in human skin fibroblasts [7] and human keratinocytes [8]. Fucoidan has showed similar inhibition of human dermal fibroblast proliferation in vitro as heparin [9]. Fucoidan-rich *F. vesiculosus* and *U. pinnatifida* extracts were shown to be effective inhibitors of elastase (IC_50_ < 100 μg/mL), tyrosinase (IC_50_ < 50 μg/mL), and collagenase [10]. Fucoidan from *U. pinnatifida* (0.3% in acetone/olive oil mixture) showed potent activity for the treatment of atopic dermatitis in the NC/Nga mice model [11]. The fucospheres containing 2% of fucoidan and 0.75% of chitosan have been used for the treatment of dermal heat burns in rabbits. The application of fucospheres lead to fast skin regeneration due to the effect of fucoidan on the migration of fibroblasts, release of growth hormones and cytokines involved in the re-epithelialization [12]. Ex vivo experiments with human skin confirmed that fucoidan limits human dermal elastic network degradation by human leukocyte elastase, and protects dermal elastic fibers against human leukocyte elastase hydrolysis [13].

Fitton et al. have shown in double-blind, placebo-controlled clinical studies that gel with 0.3% fucoidans from *U. pinnatifida* or *F. vesiculosus* is highly effective in inhibiting the erythema and water loss caused by UV-induced inflammation [10]. In patients with atopic dermatitis, it was shown that fucoidan mediates suppression of IgE in blood cells [14]. The cream containing 4% fucoidan from *Nemacystus decipiens* was found to be effective after topical application in the treatment of patients with oral herpes [15].

Thus, fucoidan has been used in transdermal formulations targeting inflammatory skin conditions, for treatment of superficial thrombosis, vascular permeability diseases, subcutaneous wounds, and burns [1]. The use of anticoagulants is one of the options for the treatment of disseminated intravascular coagulation and venous thromboembolic disease [16]. Fucoidan is a promising natural anticoagulant. It has shown significant heparin-like anticoagulant activity [17,18,19,20]. Effective inhibition of thrombin and factor Xa by fucoidan from *F. evanescens* has been described by Lapikova et al. [21]. The antithrombotic activity of fucoidan from *Saccharina latissima* has been confirmed by Ustyuzhanina et al. [22]. Recently, we have reported a significant heparin-like anticoagulant effect for an ointment with 15% of fucoidan after topical application on rats [23].

Little is known about the pharmacokinetics of fucoidan. Few papers have reported on the pharmacokinetics of this polysaccharide after peroral administration [24,25,26]. However, the pharmacokinetics of fucoidan after topical application is not described. Recently, we have developed the method of measuring the anti-activated factor X (anti-Xa) activity by an amidolytic assay and successfully applied it to the pharmacokinetics and tissue distribution of fucoidan [26]. In this study we report the pharmacokinetics of fucoidan after topical application of ointment to rats.

## 2. Results and Discussion

The topical application is considered the most convenient and comfortable way for drug delivery to patients. However, the skin is also an exceptionally effective barrier that prevents the permeation of most commercially available transdermal drugs [27,28,29]. The diffusional barrier is localized in the stratum corneum of skin, and prevents entry of molecules with molecular weight (Mw) over 350 Da [30]. Yang et al. have shown that fucoidan with Mw 10–300 kDa has exhibited the strongest anticoagulant activity [31]. Due to the relatively large Mw, negative charge and hydrophilicity fucoidans generally penetrate the skin poorly [10]. To overcome this disadvantage, we have developed an ointment formulation for fucoidan, which shows significant anticoagulant activity after topical application on rats [23]. This fucoidan, with an average Mw 750 kDa, contains 79.5% neutral carbohydrates, 27.0% sulfate residues, and 0.7% uronic acid. Transcutol was used as the penetration enhancer, and polyethylene glycol as the surfactant [32]. The anticoagulant activity of our formulation that contains fucoidan is in agreement with previously published studies, where fucoidan-possessing high sulfate, and low uronic acid content, display high anticoagulant activity [17,33]. In line with our previous studies, we considered studying the pharmacokinetics of the ointment with fucoidan (OF).

Following topical application, drugs that reach the dermis can be either systemically absorbed or continue to diffuse into underlying tissues. Drug permeability across the skin has often been regarded as the primary criterion for the efficiency of transdermal delivery without proper assessment of drug disposition in local tissues. In this study, the local tissue disposition and systemic absorption of fucoidan were investigated after topical, and intravenous (i/v) administration in rats, in order to evaluate the feasibility of transdermal delivery of fucoidan for local and systemic effects.

Single doses (50–150 mg/kg) and multiple doses (100 mg/kg during five days) of a topical application of ointment with fucoidan caused no signs of dryness, erythema, hemorrhage, edema or erosion/excoriation at application sites in rats. 

**Figure 1:**
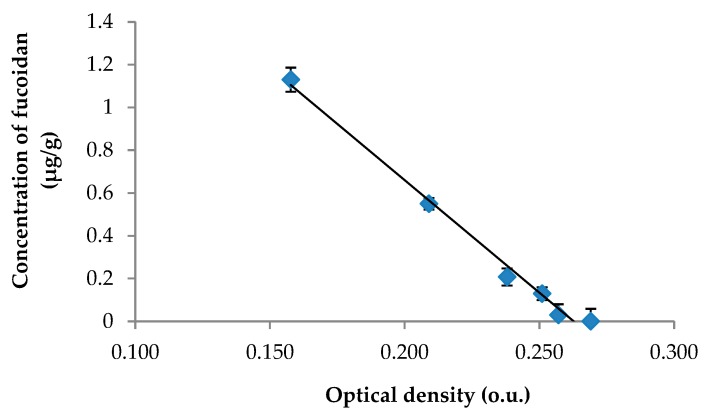
The calibration curve for the calculation of fucoidan in the skin tissue.

Based on previously published data [25], the anti-Xa activity was selected as the biomarker for our study of pharmacokinetics of fucoidan. The previously developed and validated amidolytic assay for fucoidan in rat plasma was revalidated for the skin tissue. The calibration curve for fucoidan was linear over a concentration range of 0.014–1.13 μg/g (Figure 1). The fucoidan concentration in the skin tissue was calculated according to the equation: *у* = −10.51*x* + 2.764; (*R*^2^ = 0.9904), where *х* is the optical density (o.u.) and *у* is the concentration of fucoidan (μg/g skin tissue). The validation data for the method of determining fucoidan concentration in the skin tissue are presented in Table 1.

**Figure 2:**
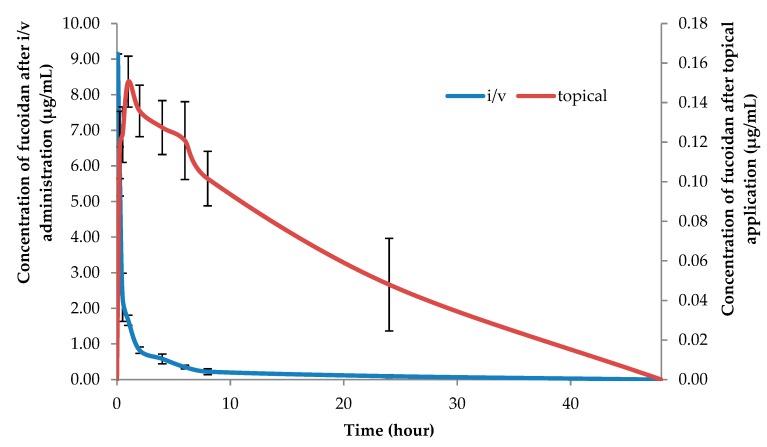
Fucoidan concentrations in plasma after i/v administration (100 mg/kg) and topical application (100 mg/kg) in rats (mean ± SD, *n* = 5).

After revalidation, the method was successfully applied for the analysis of fucoidan in plasma and tissues. The concentration-time profiles of fucoidan in plasma after i/v administration and topical application to rats are shown in Figure 2.

The plasma profile in rats after i/v administration (100 mg/kg) exhibited two exponential phases with a relatively long elimination half-life of 9.47 ± 2.34 h (Table 1). The topical application of the ointment with the same dose of fucoidan (100 mg/kg) resulted in long sustained profiles in plasma (Figure 2). Based on the concentration-time profiles data, the plasma half-life and the bioavailability of fucoidan after topical application were estimated to be 20.75 ± 9.43 h and 17.7 ± 7.7% of the applied dose, respectively. The longer half-life and low bioavailability of fucoidan after topical application was probably due to the quick drug penetration and restrain in the skin as shown in Table 2. The significant reservoir function of the skin was first discussed by Vickers [34]. However, the reservoir capacities of the skin were found to vary widely for different drugs, and substances with lower diffusivities in the stratum corneum exhibited a more prominent skin depot formation [35]. The reservoir function of the skin is an important determinant of the duration of action of a transdermal drug [36].

Table 3 shows the fucoidan concentrations in the skin and muscle tissues of the rats after topical application at the dose of 100 mg/kg. The data indicates that a significant amount of the penetrated drug is retained in the skin after the topical dose. The fucoidan concentrations in the skin during the first hour after sampling remain nearly the same, suggesting that the epidermis is saturated with the drug. However, appreciable amounts of fucoidan (0.27 ± 0.16 and 0.24 ± 0.07 µg/g tissue) were found in the 1 and 2 h muscle samples. The initially high muscle-plasma (M-P) concentration ratio of 1.18 is indicative of direct permeation of fucoidan into the muscle tissues after the topical application. Moreover, at 1 and 2 h postdose, drug concentrations in the muscle have increased, despite increased plasma concentration, resulting in the increased M-P ratios of 1.77 and 1.75, respectively. The increased fucoidan concentrations in the muscle samples could be attributed to the slow removal of the drug by the systemic circulation.

It was previously shown that oral administration of fucoidan to rats leads to a rapid increase of concentration in the stationary state one hour after administration and lasts for the next six hours. Based on the data, the elimination half-life from blood plasma, calculated on oral administration, could be estimated as 3.44 ± 1.70 h [26]. Figure 2 shows that the application of the OF resulted in long, sustained concentration-time profiles in plasma, reaching the steady state concentration at 1 h postdose and lasting for the next ~30 h. Based on the concentration-time data, the plasma half-life and the bioavailability of fucoidan after topical application (100 mg/kg) were estimated to be 20.75 ± 9.43 h and 17.7% of the applied dose, respectively. The longer half-life of the fucoidan after topical application was probably, in part, due to the large drug accumulation in the skin, as shown in the skin disposition study using rats.

It was found that the pharmacokinetics of fucoidan after topical OF application was linear in the range of 50–150 mg/kg. AUC_0-48,_ T_1/2_ and С_max_ evidently increased after the dose increased, but wide data variations were observed (Table 2). Since in the experiment with a single administration of fucoidan, the linear pharmacokinetics was established, one dose level (100 mg/kg) was used in experiments with repeated dose administration.

**Figure 3:**
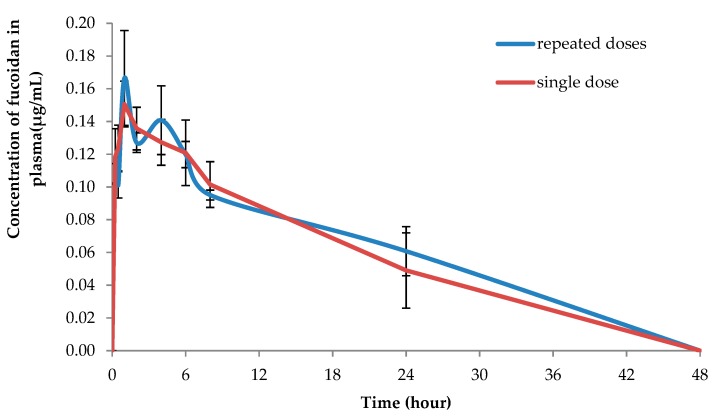
The mean plasma profiles of fucoidan after topical application of OF to the rats after single (100 mg/kg) and repeated doses (100 mg/kg during 5 consecutive days).

Figure 3 demonstrates the concentration-time profiles of fucoidan after topical application of OF in plasma after a single dose (100 mg/kg) and after repeated daily dosing with 100 mg/kg of fucoidan during five days. After the repeated dose of topical application, fucoidan exhibited higher apparent half-life of elimination (28.06 ± 7.92 h) and long circulation time (41.12 ± 10.70 h). However, the difference was not statistically significant when compared to the single dose administration, which evidenced about the absence of its accumulation in the plasma after the repeated application (Table 2). 

The topical application of formulations containing fucoidan has been reported as an effective method for the treatment of atopic dermatitis, dermal burns, oral herpes, and as an anticoagulant [11,12,15,23]. It is known that the rodent skin does not always have identical permeation properties as the human skin. In human, permeability of hydrophilic drugs is remarkably lower than that in animals [37]. It is necessary to note that systemic exposure in humans may be significantly overestimated if the risk assessment is based only on the results of an in vivo study [38]. Future studies in humans are therefore required. Since this is the first report about the pharmacokinetics of fucoidan after topical application, the present study may shed new light on the penetration of fucoidan through the skin and the rationality of transdermal formulations with this polysaccharide. 

## 3. Materials and Methods 

### 3.1. Chemicals

Fucoidan with the average molecular mass 735 kDa was obtained from MMBI KSC RAS, Murmansk, Russia. Fucoidan was extracted from *F. vesiculosus* as described previously [25]. Poloxamer 407 (Kolliphor^®^ P 407), Polyoxyl 40 hydrogenated castor oil (Kolliphor^®^ RH40), polyethylene glycol 400 (Kollisolv^®^ PEG 400) were provided by BASF (Ludwigshafen, Germany). Extra-virgin olive oil was purchased at a local market. Diethylene glycol monoethyl ether (Transcutol^®^ P) was a gift from Gattefossé (Saint-Priest, France). Acepromazine (Fermenta Animal Health Co., Kansas City, MO, USA), ketamine (Fort Dodge Laboratories, Inc., Fort Dodge, IA, USA), xylazine (Miles Inc., Shawnee Mission, KS, USA), and other chemicals were used as received from the suppliers.

### 3.2. Animals

Male outbred rats (*n* = 250) were obtained from Rapplovo animal house (St. Petersburg, Russia). The animals were kept under standard conditions with a 12-h light–dark cycle, at an ambient temperature (22 ± 2 °C), and relative humidity of 60 ± 10%. They had free access to food (Standard diet: Volosovo, Russia) and water ad libitum. Rats (*n* = 5 per time point) fasted overnight before the experiment. Rats were divided into five groups: group A, intravenous (i/v) injection of fucoidan (single dose, 100 mg/kg) for determination absolute bioavailability; groups B, C and D, topical application of 15% fucoidan ointment (single dose of 50, 100 and 150 mg/kg, respectively); and group E, topical application of 15% fucoidan ointment (100 mg/kg, once a day) during 5 days. The rationality of this dose selection is supported by a previous report [26]. Four groups were treated with the 15% fucoidan ointment on the dorsal site (3 cm × 3 cm) for sampling at different time points (15, 30 min, 1, 2, 4, 6, 8, 24, and 48 h postdose). One day prior to dosing, the ventral and dorsal areas of the skin were carefully shaved with an electric clipper (Oster, Model A-2, Milwaukee, WI, USA) under light anesthesia using intraperitoneal injection of a mixture containing ketamine and xylazine (10:1 v/v, 50 mg/mL). No signs of skin damage were observed after shaving. Regarding application, 350, 700 and 1000 milligrams of the 15% fucoidan ointment was applied uniformly on the marked area of the dorsal skin, approximately 3 cm × 3 cm, and allowed to dry. After application, the rats were euthanized in a CO_2_ chamber at different time points (15, 30 min, 1, 2, 4, 6, 8, 24, and 48 h), and the blood was collected by cardiac puncture. The blood was transferred to sodium citrate tubes, centrifuged at 3000× *g* for 15 min at 4 °C, and then the plasma was collected and stored at −20 °C. 

The skin and muscle were cut into small pieces to determine the amount of fucoidan in the viable skin (epidermis and dermis) and muscles. Prior to excising tissue samples, the dosed area of the skin was completely washed with wetted cotton in 50% ethanol. Each tissue sample was precisely weighed and homogenized (Polytron PT-MR 1600E, Kinematica AG, Lucerne, Switzerland) in 0.15 μM Tris-HCl buffer (pH 8.4). After vortex mixing and centrifugation for 15 min at 3000× *g* (EBA21 tabletop centrifuge, Hettich, Westphalia, Germany), the upper phase was collected and used for an amidolytic assay.

An aqueous solution of fucoidan (10 mg/mL) at the doses of 100 mg/kg was used for the i/v administration to rats. After administration, the rats were euthanized in a CO_2_ chamber at the time points of 5, 15, 30 min, 1, 2, 4, 6, 8, 24, and 48 h. The animals from group E were euthanized on the 5th day of experiments. The blood was collected in sodium citrate tubes by cardiac puncture, centrifuged at 3000× *g* for 15 min at 4 °C, plasma was collected and stored at −20 °C.

Experiments were performed according to the directive 267, “Regarding the statement of regulation of laboratory practice of the Ministry of Health of the Russia” (2003) and the EEC Directive of 1986 (86/609/EEC), and were approved by the Ethical Commission of the St. Petersburg Institute of Pharmacy (Leningrad Region, Vsevolozhsky District, Kuzmolovo P 245, Russia).

The ointment was prepared as described previously [23]. Shortly after, an appropriate amount of poloxamer was slowly added to water, and the mixture was left in a refrigerator until becoming a clear solution. Fucoidan and Transcutol were dissolved in a water solution of poloxamers and then mixed into the cold solution. The solution was incubated at room temperature until a homogeneous gel was formed. Separately, olive oil, kolliphor and PEG 400 were mixed at room temperature. Then a fucoidan phase was added to the oil phase with vigorous stirring.

### 3.3. Analysis of Fucoidan in Plasma and Tissue

The amount of fucoidan in plasma and tissues was determined by the amidolytic assay using ReaChrom Heparin kit (Renam, Russia) as described previously [26]. Optical density was recorded at 405 nm on a microplate spectrophotometer X-Mark (Bio-Rad, Hercules, CA, USA). The endogenous level of products reacted with the heparin kit was subtracted at each time point in each sample. The method was validated according to the International Conference on Harmonization (ICH) guidelines [39,40]. 

### 3.4. Pharmacokinetic and Statistical Analysis

A PKSolver add-in for the Excel was used for the pharmacokinetic calculations of fucoidan in tissues and plasma. The parameters were calculated from the concentration-time data using a noncompartmental pharmacokinetic model as described previously [26]. The results are expressed as the mean ± standard deviation (SD) (*n* = 5 for each time point). 

The bioavailability of fucoidan from the ointment was calculated by comparing the area under the concentration-time curve for topical dose (AUC_t_, 0–48 h) and that for i/v dose (AUC_iv_, 0–48 h). The Student *t*-test was used for the test of statistical difference and *p* values of less than 0.05 were considered significant.

## 4. Conclusions

Although there is limited available literature, research on the fucoidan use for transdermal therapy and its pharmacokinetics has been increasing. Our results indicate that fucoidan in ointments penetrate the skin barrier and accumulate in the striated muscle. Our results support the rationality of the topical application of fucoidan formulations. 

## Figures and Tables

**Table 1:** The validation data for the method determining fucoidan in the skin tissue.

Parameter	Range
Accuracy, *%*	
ULOQ (1.13 μg/g)	0.6–3.4
Middle-quality control (0.56 μg/g)	1.7–9.7
Low-quality control (0.14 μg/g)	9.2–14.8
LLOQ (0.014 μg/g)	2.9–12.3
Intraday/Interday precision (RSD), *%*	
ULOQ (1.13 μg/g)	0.9–1.9/3.5
Middle-quality control (0.56 μg/g)	3.4–6.1/7.6
Low-quality control (0.14 μg/g)	2.3–5.0/6.8
LLOQ (0.014 μg/g)	3.7–4.7/10.9
LOD, μg/g	0.0035

ULOQ, upper limit of quantification; LLOQ, lower limit of quantification; LOD, limit of detection.

**Table 2:** Pharmacokinetic parameters of fucoidan after single and repeated dose administration to rats.

Sample	Dose	Parameter				
	(mg/kg)	AUC_0–48_ (µg·h/mL)	MRT (h)	T_1/2_ (h)	C_max_ (μg/mL)	T_max_ (h)
Single dose						
Plasma, i/v	100	10.83 ± 0.32	8.08 ± 1.92	9.47 ± 2.34	9.15 ± 0.60	-
Plasma, topical	50	0.74 ± 0.11	20.99 ± 10.74	14.41 ± 7.52	0.12 ± 0.02	1.20 ± 0.44
Plasma, topical	100	1.92 ± 0.94	30.20 ± 13.70	20.75 ± 9.43	0.15 ± 0.01	1.00 ± 0.00
Plasma, topical	150	3.08 ± 0.21	29.94 ± 4.51	20.60 ± 3.07	0.18 ± 0.01	1.20 ± 0.01
Skin*, topical	100	0.94 ± 0.11	9.00 ± 4.36	6.28 ± 3.46	0.27 ± 0.01	0.25 ± 0.00
Striated muscle*, dermal	100	2.22 ± 1.18	16.15 ± 4.40	10.64 ± 9.94	0.31 ± 0.12	1.80 ± 1.30
Repeated doses (5 days)						
Plasma, topical	100	2.10 ± 0.69	41.12 ± 10.70	28.06 ± 7.92	0.18 ± 0.05	1.10 ± 0.55

* AUC_0–48_ (μg·h/g) for tissues; C_max_ (μg/g) for tissues. AUC_0–48_, the area under the curve; MRT, mean residence time; T_1/2_, apparent half-life of elimination. The results are expressed as the mean ± SD (*n* = 5).

**Table 3:** Fucoidan concentrations (µg fucoidan/ g tissues or mL plasma) following topical application (100 mg fucoidan /kg weigh rat) of ointment (Mean ± SD).

Tissues	15 min	30 min	1 h	2 h	4 h	6 h	8 h
Skin	0.274 ± 0.013	0.231 ± 0.028	0.224 ± 0.029	0.138 ± 0.032	0.105 ± 0.010	0.092 ± 0.008	0.050 ± 0.046
Muscle	0.141 ± 0.033	0.162 ± 0.049	0.268 ± 0.156	0.238 ± 0.067	0.196 ± 0.053	0.150 ± 0.037	0.115 ± 0.043
Plasma	0.119 ± 0.017	0.124 ± 0.014	0.151 ± 0.013	0.136 ± 0.013	0.127 ± 0.012	0.121 ± 0.019	0.102 ± 0.014
M-P ratio	1.18	1.31	1.77	1.75	1.54	1.24	1.13
